# Students' attitudes towards the introduction of a Personal and Professional Development portfolio: potential barriers and facilitators

**DOI:** 10.1186/1472-6920-9-69

**Published:** 2009-12-01

**Authors:** Sarah Ross, Alison Maclachlan, Jennifer Cleland

**Affiliations:** 1 University of Aberdeen, Division of Medical and Dental Education, Polwarth Building, Foresterhill, Aberdeen, AB25 2ZD, UK

## Abstract

**Background:**

Portfolios, widely used in undergraduate and postgraduate medicine, have variable purposes, formats and success. A recent systematic review summarised factors necessary for successful portfolio introduction but there are no studies investigating the views of students inexperienced in portfolio use towards portfolio learning. This study's aim was to survey student views about a prospective Professional and Personal Development (PPD) portfolio.

**Methods:**

This was a qualitative, focus group study. All focus groups were taped and transcribed verbatim, and anonymised. The transcripts were analysed inductively, using framework analysis.

**Results:**

Four focus groups were carried out with 32 undergraduate medical students naïve in portfolio use. Three themes relevant to portfolio introduction emerged. The first theme was the need for clear information and support for portfolio introduction, and anxieties about how this could be supported effectively. The second was that students had negative views about reflective learning and whether this could be taught and assessed, believing formal assessment could foster socially acceptable content. The third was that participants revealed little understanding of reflective learning and its potential benefits. Rather portfolios were seen as useful for concrete purposes (e.g., job applications) not intrinsic benefits.

**Conclusion:**

Undergraduate medical students without experience of portfolios are anxious about portfolio introduction. They require support in developing reflective learning skills. Care must be taken to ensure students do not see portfolios as merely yet another assessment hurdle.

## Background

Doctors are encouraged to be reflective practitioners and many maintain a portfolio [1]. Many postgraduate trainees in the UK keep a portfolio and many medical schools have introduced portfolios as a component of learning and assessment.

The format and purposes of portfolios across undergraduate and postgraduate medicine vary widely. For example, portfolios may be limited to particular subjects such as communication skills [2] or personal and professional development [3]. A portfolio may be used in summative assessment [4] or for formative purposes only. Portfolios may be "information collected in evidence of the owner's learning process and/or competence levels" or concerned with "reflections on educational achievement and personal and professional development", or both [5].

The Best Evidence Medical Education Collaboration has produced a definition which encompasses all types of portfolio used in Medical Education:

"A collection of evidence of student activity (whether paper-based or electronic) that:

• Outlines the student's own learning experience (e.g. patients seen, study subjects covered, articles read)

• AND Requires some "intellectual processing" on the part of the student

• AND Draws together more than one item, clinical case, task, report, reflective task, etc

• OR Is a learning journal, a collection of student reflections on their learning" [6]

However, perhaps as a result of the aforementioned variety of uses, the success of portfolio implementation and use is highly variable [7]. Driessen's recent systematic review of the factors which increase success of a portfolio include:

• a proper introduction and mentoring;

• integration within context and procedures;

• provision of information to students and teachers;

• provision of clear guidelines that do not curtail students' freedom;

• user-friendliness that includes limited time demands on students and mentors [7].

Strong leadership and faculty support for portfolio implementation and use are also vital [5,8].

While Driessen's review provides much useful information for medical schools who are attempting to implement a portfolio [7], other questions about portfolio implementation and use remain unanswered. Undergraduate medical student views of how best to introduce a portfolio are unknown, but these are also likely to be relevant to successful implementation and effectiveness of portfolios [9]. This may be particularly important when the aims of a portfolio explicitly include promoting adult learning styles [10] and encouraging reflective learning [11,12], both of which may involve changing student attitudes. The relative inexperience of undergraduate students may influence their attitudes towards, and gains from, portfolio learning. For instance, undergraduate medical students may be unskilled at reflective practice (as seen in dental students [13]) and may be less likely to recognise the benefit of a portfolio.

Our study aimed to answer the research question: how can a PPD portfolio be best implemented in an undergraduate medical degree? The context in which the study took place was a medical school which was planning the implementation of a Personal and Professional Development (PPD) formative portfolio.

## Methods

In the absence of published UK empirical work on student views of portfolio implementation, we selected qualitative methodology to help identify the issues which need to be addressed to effectively implement a PPD portfolio. We chose focus groups as these are not only able to generate data regarding perceptions and beliefs, but can also be particularly useful as the group setting can make people more confident in sharing information [14]. Further, focus groups allow participants to ensure that the topics under discussion are directed by participants themselves, not just by researchers' agendas [15].

### Participants

All medical students from the University of Aberdeen studying or working locally at the time of the study (n = 53) were invited to take part via an email circular from the Medical School office. The dates and times of the focus groups were set in advance of the invitation going out, organised for lunchtimes for ease of participation. Those who expressed interest in the study were then contacted by the researchers to confirm attendance at specific focus groups.

The North of Scotland Research Ethics Committee stated that ethics permission was not required for this project as it involved healthy, adult volunteers. However, in the interests of ethical quality assurance, care was taken to provide appropriate written information so students could make an informed decision to participate or not. Written, informed consent for data collection and publication of anonymised data was obtained from all participants.

### Data collection

Four focus groups were held in July and August 2008. All focus groups were held in the University of Aberdeen School of Medicine and Dentistry.

The same semi-structured discussion guide was used for all groups. This was developed through a literature review. The topics covered included: understanding of reflective learning; use of a portfolio; potential facilitators and barriers to portfolio use; content; format and student support. The focus group discussions were lead by JC, who has experience in qualitative, focus group research [16], and co-facilitated by AM. Participants were provided with a certificate of participation.

### Analysis

All focus groups were taped and transcribed verbatim, and anonymised. The transcripts were analysed inductively, using framework analysis [17], during which we determined content-related themes (what participants said). Care was taken to ensure agreement and disagreement were vocalised by the participants as non-verbal communication (e.g., nods of agreement) cannot be identified from recordings at the time of analysis. Framework analysis was used to classify and organise the data according to key themes (main and sub-themes), concepts and emergent categories, which were used to examine the data for patterns and connections. Process-related themes such as the use of language and humour were not analysed.

Focus group transcripts were initially analysed independently by each author who developed categories or themes as they emerged from the data [18]. All authors then met to discuss and examine these independent analyses using the constant comparative method, where items were compared and contrasted, and themes negotiated and checked to establish analytical categories, or the "fit" between items, and to group data into categories and sub-categories. Data analysis was managed using a paper (rather than a software) system.

## Results

Of 53 students available at the time of the project, 32 agreed to participate in the focus groups (60%). They represented each year of study other than 5^th ^year: due to the timing of the study, 5^th ^year students had finished their MBChB.

The focus group discussions ranged from 40-60 minutes in length.

Three main themes relevant to the task of effectively introducing a PPD portfolio, emerged. These are set out below.

### Information and support needs

The data indicated that most students wanted a clear explanation of the purpose of a portfolio, in a format which incorporated opportunity for questions, such as a lecture or tutorial. Written guidance was also seen as essential to supplement the introduction of a portfolio, and several students said the content should include worked examples and links to further resources: "I think if you had an example of a filled in portfolio, kind of the good, the bad and the ugly..."

Student views on the format of a portfolio were mixed. Some students preferred a traditional paper portfolio that could be viewed in its entirety, while others wanted an online platform which would provide reminders as to completing portfolio tasks (and which would not get lost): "I appreciate something in front of me, rather than online. But then I can see the advantages of having something online so you can go straight to wherever it is you want to go to."

The majority of students were anxious about writing reflectively and wanted support in this, and feedback on early reflective exercises in order to improve their skills, if required, for future exercises.

Students felt that tutor-led small group sessions for portfolio support would be useful if these facilitated honesty and openness about their experiences. However, there were some concerns about competitiveness, group interaction and the potential for inequality in group contributions raised by a minority of students. For example, "We're all in competition with each other for careers, that sounds horrible but that's what it's like so I don't think many people would be willing to contribute much to that kind of group work." "I just annoys me if you look at all the people in my class, who're always late and never bother turning up, why should I sit there and help them?"

Tutor characteristics were consistently reported as important to students: they should be enthusiastic and have experience of using a portfolio themselves.

"Think it would have to be people who wanted to actually be involved."

"Someone who has kept a really good portfolio themselves, who wants to do it."

Use of peer-led (e.g., older students) tutorials was viewed less positively: "I don't know how much experience they would have compared to a doctor...."

There was some concern from many students about the potential workload involved in a portfolio and how this would be integrated with their other commitments. Assessment of a portfolio was also a concern (as yet another method of assessment) but, on the other hand, some form of assessment was seen as motivating.

"I don't think reflection's something you can assess, y'know like an exam, you can assess someone's memory, test their memory but I don't think you can test reflection. Quite a personal thing." "At least that ensures that students will take it seriously."

However, some participants discussed how assessment of portfolio content may encourage students to write socially acceptable or perceived "correct" answers.

"Again with the portfolio thing you are just going to end up saying, become good at writing essays which is exactly what they want to hear it I think."

A pass/fail system where a fail would constitute a poor effort as opposed to a judgement of the content, was the favoured method of assessment from the majority of participants: "But people who've made an absolute shocking or shabby attempt at it..." "Cause as it is all quite personal things, can't say that's wrong, right or wrong."

### Reflective learning

Students found it difficult to provide a definition of reflective learning. While one or two participants were able to articulate the need to think about learning experiences (Schon's reflection on action [19]), no one revealed a deep understanding of the process and its potential benefits.

"It's learning from your mistakes. But it's also learning from what you've done well and why."

Indeed, many comments were negative with some asking whether this was a skill that could be taught at all and others suggesting that formalised reflection was inappropriate.

"I think it's something that you do off your own back; it's not really something that you can integrate into a course." "I don't know if you can really teach people how to reflect. Surely you just do that."

For example, several students commented that reflection on an individual task was done almost unconsciously: "...in a real life situation if you make a mistake, if you I don't know prescribe the wrong drug or y'know harm a patient, you're going to reflect on that anyway, if you're a competent doctor and learn from your mistakes so I don't find it particularly useful writing down." Whilst a few students recognised some potential value in reflection, some students felt that they would face difficulties in reflecting, particularly worrying that they may become over-critical of themselves.

"It's not necessarily good though if you're the type of person who's really hard on themselves because like, you're not going to, like, benefit from it, it's just going to make you doubt yourself, even more than you already do." When this was explored further, they discussed how developing such skills as an undergraduate would be of use when they came to work as a foundation doctor.

"I got the impression from the start that portfolios were just for practice of doing portfolios before we actually have to do them for our job."

### Benefits of the portfolio

Students were then given an example of portfolio content to illustrate some of the potential benefits. The potential advantages in having skills and material to aid in completing foundation programme application were widely perceived as important.

"I agree if it was emphasised from the beginning, you know this is going to be helpful and you know you will need to answer these type of questions when you're applying for your first post, I think people would spend more time on it and it would be sort of seen that there's a purpose to it."

Other benefits of the portfolio included the view that a portfolio would prepare the students for when they came to use portfolios in future (when working as a doctor) and that they could use the portfolio as a useful record of non-academic achievements such as extra-curricular activities. For example, "It's good to get into the mind-set because we'll have to do this, I assume, all the way through." Overall, benefits were perceived in terms of concrete outcomes such as job applications or making post graduate life easier. Understanding of portfolio based learning as a learning tool and for personal development was lacking.

## Discussion

We have identified that undergraduate medical students who are naïve in portfolio use know little about reflective learning or the use of portfolios to support such reflective learning. The need for introductory information and support was clearly identified as students were anxious about the introduction of a new method of learning, meaning yet more work and one more thing to be assessed. Portfolio learning support, and portfolio assessment, were not seen as straightforward by participants with competitiveness and social acceptability discussed as potential issues. While students could begin to see the potential benefits of reflective portfolio learning, this insight tended to be limited to seeing concrete outcomes such as a record of examples which would help them in completing foundation programme applications.

A number of the findings identified in this study supported previously published data such as the need for appropriate information, guidance and support as well as a user-friendly format and a concern about portfolios being overly time-consuming [5,13]. However, our empirical approach adds to existing knowledge in two main ways.

Firstly, the lack of understanding of reflective learning in our participants was particularly striking. While this may be partly explained by their lack of experience of this learning method at medical school, it was clear that negative attitudes and misconceptions existed about reflective learning. This may reveal a wider view amongst medical students and doctors: Grant et al felt a lack of understanding of how reflective learning related to the medical curriculum limited the number of students willing to participate voluntarily in a study of reflective learning [20]. Pee reported findings from dental education that both tutors and students found reflection difficult and did not prioritise it, with some students reporting a dislike of the process [13]. In the postgraduate medical setting, Pearson and Heywood's study of the use of portfolios by GP registrars reported that the "majority of registrars did not use the portfolio in reflection; many considered it unhelpful and many were unenthusiastic" [21]. Together these findings indicate that there may be a culture in medicine which does not support reflective learning, despite its increasing popularity in medical education at all levels. This paradox clearly warrants further exploration as learners are unlikely to gain from a process that they do not value. However, the results of this study suggest that introducing and supporting portfolio learning in student-centred way, and ensuring that tutors model appropriate attitudes in order for reflective learning to reach its potential, may go some way to supporting the development of positive attitudes towards portfolio and reflective learning. This will require careful planning and evaluation of the process of implementation.

Secondly, the misperception of reflective learning was further illustrated by participant focus on its uses for training post applications. While some students were aware of the requirement for lifelong reflective learning, most seemed very uncertain why this is the case. A clear explanation of learning styles and the intrinsic benefits of reflection for lifelong learning are an obvious prerequisite for a portfolio. However, there is a potential danger that emphasising the similarities between portfolios and application forms, as well as the likely use of portfolios and reflective learning in professional revalidation, would add weight to the pragmatic approach voiced by the participants rather than to a genuine understanding of lifelong learning [20].

Another surprising finding was that, although some students were keen on small group support for portfolio reflective learning [22,23], a significant proportion of participants seemed concerned about competition between students in such sessions. This may be due to the perception that the portfolio is solely concerned with preparation for foundation programme applications. Again, it reveals a fundamental lack of understanding about the learning process.

One potential weakness of this study is that the data were collected in one geographical locality, and one medical school, with participants from a limited pool of available students. Therefore, student participants were not representative of all year groups. This may have contributed to the similarity in views within and between groups. However, the response rate to invitations to participant in the study was high, suggested an intrinsic interest in the topic. Furthermore, all our participants had experience of studying or working together, and seemed comfortable discussing their views and experiences. Replicating this study in diverse locations, or carrying out similar work with students who have experience of using a portfolio may be helpful in illuminating the generalizability of these findings. Further work could explore views of portfolios and reflective learning in students for whom these forms of learning are an integral component of their undergraduate degree - do these students have a different view of reflective learning/portfolios than "naïve" students? One would hope so. The perception of reflective learning and its place in medical culture may also warrant further exploration.

## Conclusion

In conclusion, the results of this study provide useful information to inform the optimal introduction of PPD portfolios aimed at supporting medical students in reflective learning.

## Competing interests

The authors declare that they have no competing interests.

## Authors' contributions

SR conceived the study. SR and JC designed the study. AM and JC undertook the focus groups and collected the data. All authors were involved in the analysis and interpretation. All authors were involved in the drafting and revision of the paper. All authors read and approved the final manuscript.

## Pre-publication history

The pre-publication history for this paper can be accessed here:

http://www.biomedcentral.com/1472-6920/9/69/prepub
